# Gelatin-Based Zinc-Loaded Hydrogels Constructed with the Assistance of Sodium Alginate and Zinc Sulfate Solution Soaking Method

**DOI:** 10.3390/foods14213642

**Published:** 2025-10-24

**Authors:** Hongrui Chen, Xi Guan, Xianglin He, Qing Zhang, Xingzhong Zhang, Hai Chi, Zhenju Jiang, Jie Tang

**Affiliations:** 1 School of Food and Bioengineering, Food Microbiology Key Laboratory of Sichuan Province, Chongqing Key Laboratory of Speciality Food Co-Built by Sichuan and Chongqing, Xihua University, Chengdu 611130, China; 0120220038@mail.xhu.edu.cn (H.C.);; 2 Department of Chemistry, School of Science, Xihua University, Chengdu 611130, China

**Keywords:** zinc, hydrogel, delivery system, pH sensitivity

## Abstract

Constructing a zinc delivery system is crucial for scientific zinc supplementation. In this study, gelatin-based zinc-loaded hydrogels were constructed with the assistance of sodium alginate and a ZnSO_4_ solution soaking method. The zinc loading capacity, texture properties, rheological properties, microstructure, and pH sensitivity of hydrogels under different ratios of gelatin to sodium alginate were investigated. Results showed that the loading of zinc by hydrogel was successfully achieved through a ZnSO_4_ solution soaking method, and increasing the ZnSO_4_ concentration was conducive to zinc loading and hydrogel structure strengthening. Adding sodium alginate further enhanced the zinc loading capacity of hydrogel. When the concentration of ZnSO_4_ was 25 wt%, the zinc loading of hydrogel containing only gelatin and hydrogel with a 7:3 ratio of gelatin to sodium alginate was 29 mg/g and 52 mg/g, respectively. In addition, sodium alginate also endowed the hydrogel with a certain pH sensitivity. When the ratio of gelatin to sodium alginate was 7:3, the hydrogel showed obvious pH response behavior. Spectroscopy results revealed that zinc sulfate strengthened the hydrogel structure by inducing hydrophobic interactions and the formation of hydrogen bonds, while Zn^2+^ was bound to oxygen atoms through coordination bonds in hydrogel. These results could provide new ideas for the construction of zinc-loaded hydrogels.

## 1. Introduction

Zinc, as an essential micronutrient for the human body, is an important component of transcription factors and enzymes, and has multiple significant physiological functions [1]. Zinc is required for DNA synthesis, which contributes to the division and proliferation of human cells and promotes the growth and development of human body. Zinc can also enhance appetite, protect vision, and play an important role in maintaining normal immune function in the human body [2,3]. However, it is reported that about 17% of the global population is still at risk of zinc deficiency, especially in developing countries [4].

Zinc supplementation can effectively prevent zinc deficiency. It is well known that the main site for zinc absorption is the small intestine. However, Zn^2+^ is unstable in the gastric environment and readily combines with gastric acid in the body; directly taking zinc sulfate can irritate the gastrointestinal tract and cause side effects such as nausea and vomiting [5]. Therefore, an increasing number of studies have focused on the construction of Zn^2+^ delivery carriers, such as the preparation of polysaccharide chelated zinc and polypeptide chelated zinc using polysaccharides or peptides as carriers, or the preparation of food-derived nanoparticles as Zn^2+^ delivery carriers. However, the preparation steps of these carriers are overly complicated and difficult to control, and their zinc loading capacity is limited [4]. Therefore, it is highly necessary to construct new Zn^2+^ delivery carriers.

Hydrogels have a porous, hydrophilic, three-dimensional network structure, which allows them to bind to and wrap nutrients [6]. Hydrogels made from natural polymers such as proteins or polysaccharides can be obtained simply by heating or cooling after the raw materials are dissolved. pH-sensitive hydrogels are a type of hydrogel with targeted delivery properties. Their structure shrinks or swells in response to changes in environmental pH, thereby protecting or releasing the nutrients encapsulated within under specific pH environments [7,8]. Constructing a hydrogel with small intestinal pH swelling characteristics seems to be a suitable carrier for enhancing the stability of Zn^2+^ in the gastric environment.

Gelatin is the denaturation product of collagen with good gelling properties, which is commonly used for preparing hydrogels [9]. However, the hydrogel structure prepared solely from gelatin is prone to disintegration, resulting in the rapid release of inclusions [10]. In addition, these pure gelatin hydrogels do not have pH sensitivity, hardly achieving targeted delivery characteristics. Sodium alginate is a kind of natural polysaccharide extracted from kelp, giant algae, or brown algae. The molecule of sodium alginate is composed of β-D-mannuronic acid and α-L-glucuronic acid connected by (1→4) bond [11]. A sodium alginate molecule contains a large amount of -COO-, which can be protonated to form -COOH in an acidic environment, and -COOH deprotonation on the molecular chain is realized with an increase in pH, endowing sodium alginate with high pH sensitivity [12,13]. Therefore, sodium alginate can be considered for adding to gelatin solution. The interaction between sodium alginate and gelatin strengthens the hydrogel structure while endowing the hydrogel with a certain pH sensitivity. In addition, according to previous studies reported in the literature, the loading of nutrients in hydrogel delivery systems is usually achieved by directly adding nutrients to the hydrogel precursor solution, and then forming a hydrogel through heating or cooling to encapsulate the nutrients [14]. However, in a system where sodium alginate is present, directly adding Zn^2+^ to the pre-gel solution is prone to causing aggregation and precipitation, affecting the formation of hydrogel and the stability of the hydrogel structure. In our previous research, it was found that soaking gelatin-carrageenan hydrogel in K_2_SO_4_ solution can not only achieve the loading of K^+^, but also the hydrophobic interaction between the gelatin molecular chains induced by SO_4_^2−^ further strengthens the hydrogel structure, which prevents the rapid disintegration of hydrogel structure [15].

Based on the above theories, our hypothesis is that a structurally strengthened pH-sensitive zinc-loaded hydrogel can be prepared by introducing sodium alginate into gelatin hydrogel and then soaking the gelatin-sodium alginate hydrogel in zinc sulfate solution. The Zn^2+^ loading capacity, texture properties, rheological properties, structural properties, and pH sensitivity of zinc-loaded hydrogel were measured under different proportions of gelatin and sodium alginate. The results could provide new ideas for the construction of Zn^2+^ delivery systems and a theoretical basis for the development of new zinc supplementation preparations.

## 2. Materials and Methods

### 2.1. Materials and Chemicals

Gelatin (GE) (Tilapia skin, Type A, 250 Bloom) was purchased from Shanghai Xinxi Biotechnology Co., Ltd. (Shanghai, China). Sodium alginate (ALG) was procured from Shanghai Titan Scientific Co., Ltd. (Shanghai, China). Zinc sulfate heptahydrate was provided by Tianjin Fuchen Chemical Reagent Co., Ltd. (Tianjin, China). Nitric acid was purchased from Chengdu Kingsoft Chemical Reagent Co., Ltd. (Chengdu, China). Phosphate-buffered saline was procured from Beijing Lanjieke Technology Co., Ltd. (Beijing, China).

### 2.2. Preparation of Gelatin-Sodium Alginate (GE-ALG) Hydrogels

First, gelatin and sodium alginate powder were weighed separately, followed by adding a predetermined volume of deionized water for mixing. The mixture was then heated and stirred in a magnetic stirring water bath (SHJ-6AB, Changzhou Jintan Liangyou Instrument Co., Ltd., Changzhou, China) at 65 °C for over 60 min to ensure the complete dissolution and homogeneous mixing of the components. Subsequently, the solution was centrifuged at 4000 rpm for 10 min to eliminate any entrapped air bubbles. Finally, the solution was poured into cylindrical molds with a diameter of 20 mm and stored in a refrigerator at 4 °C for 12 h to obtain GE-ALG hydrogels with different ratios of gelatin to sodium alginate (GE:ALG = 10:0, 9:1, 8:2, 7:3).

### 2.3. Preparation of Zinc-Loaded Hydrogels

**Figure 1:**
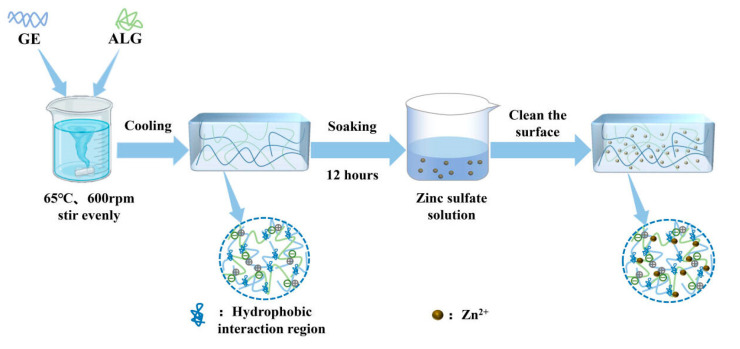
Schematic representation of the hydrogel preparation process.

To prepare zinc-loaded hydrogels, GE-ALG hydrogels were carefully trimmed into cylindrical shapes with a height of 10 mm and a diameter of 20 mm. These trimmed hydrogels were subsequently immersed in 15 mL zinc sulfate solutions with concentrations of 5 wt%, 15 wt%, and 25 wt% for 12 h to facilitate Zn^2+^ loading. The surface of each zinc-loaded hydrogel was rinsed three times with deionized water to remove any unbound ions after immersion. Finally, these hydrogels were stored at 4 °C for 12 h to ensure complete stabilization for subsequent testing. For the convenience of subsequent discussions, GE-ALG hydrogels at different gelatin-to-sodium alginate ratios and zinc-loaded hydrogels were abbreviated as xGE-yALG and xGE-yALG-z, where x, y, and z represent the concentrations of gelatin, sodium alginate, and zinc sulfate solutions, respectively. The schematic representation of the hydrogel preparation process and the formula of each hydrogel are shown in Figure 1 and Table 1, respectively.

### 2.4. Zn^2+^ Loading Capacity (LC)

The Zn^2+^ LC was determined using flame atomic absorption spectrometry (FAAS) (AA-7800, Shimadzu Instruments Co., Ltd., Suzhou, China) according to the method of Yaman & Kaya [16] with some modifications. Zinc standard solution was introduced into the flame atomizer and their absorbance values were measured at 213.8 nm to construct the calibration curve. About 0.5 g of the zinc-loaded hydrogel was accurately weighed into a microwave digestion vessel, and then 7 mL of nitric acid was added for microwave digestion. Then, the digestion solution was diluted to an appropriate concentration to determine the Zn^2+^ content in different hydrogels.

### 2.5. Texture Profile Analysis (TPA)

Texture was determined according to the method of Xie et al. [17] with some modifications. The texture properties of zinc-loaded hydrogel samples, including hardness and springiness, were evaluated using a texture analyzer (TA-XTPLUS, Xiamen Chaotech Instrument Co., Ltd., Xiamen, China) under TPA mode. The TPA test parameters were set as follows: P/36R cylindrical probe, pre-test speed of 3 mm/s, test speed and post-test speed of 1 mm/s, compression distance of 5 mm, and holding time of 5 s.

### 2.6. Rheological Properties

The rheological properties of zinc-loaded hydrogels were obtained using the rheometer (Anton Paar MCR 302, Chengdu Century Ark Technology Co., Ltd., Chengdu, China) equipped with a parallel plate 20 mm in diameter according to the method of Chen et al. [15]. Prior to measurements, dynamic strain sweep was performed to identify the linear viscoelastic region. Subsequently, a dynamic frequency sweep test was carried out at 25 °C within the frequency range of 0.1 to 100 Hz and at a constant strain of 1%, which was in the linear viscoelastic region. The variations in storage modulus (G′) and loss modulus (G″) over this frequency range were recorded to evaluate the structural characteristics of zinc-loaded hydrogels.

### 2.7. Melting Temperature and Gelling Temperature

The melting temperature and gelling temperature of zinc-loaded hydrogels were also evaluated using the rheometer (Anton Paar MCR 302, Chengdu Century Ark Technology Co., Ltd., Chengdu, China) equipped with a parallel plate 20 mm in diameter. Temperature scanning mode was adopted, and the strain and frequency were fixed at 1% and 1 Hz, respectively. A hydrogel disc with a diameter of 20 mm and thickness of 1 mm was placed in a 1 mm gap between parallel plates. To determine the melting temperature, temperature was increased from 25 °C to 60 °C at a rate of 5 °C/min, during which the changes in storage modulus (G′) and loss modulus (G″) with temperature were recorded [18]. Subsequently, the temperature was decreased from 60 °C to 4 °C at a rate of 8 °C/min, and the variations in G′ and G″ were again recorded to determine the gelling temperature. Throughout the entire experiment, dimethyl silicone oil was applied around the sample to prevent water evaporation [19].

### 2.8. pH Sensitivity

The zinc-loaded hydrogels were cut into thin slices and subsequently immersed in 30 mL of PBS solutions with pH values of 2 and 7 for 12 h to allow hydrogels to fully swell. Images of these hydrogels were captured before and after swelling, and the pH sensitivity of the hydrogels was assessed based on the morphology changes in hydrogels in different pH solutions.

### 2.9. Microstructure

The morphology of hydrogel samples was measured using a field emission scanning electron microscope (ZEISS Gemini 300, Carl Zeiss AG, Oberkochen, Germany). First, the freeze-dried zinc-loaded hydrogel sample was adhered to the platform using conductive adhesive [20]. The morphology was observed at a voltage of 5 KV and a magnification of 50× after spraying with gold for 45 s.

### 2.10. Fourier Transform Infrared Spectra (FTIR)

A Fourier transform infrared spectrometer (L1600400, PerkinElmer Instruments Ltd., Waltham, MA, USA) was used to record the FTIR spectrogram of hydrogel samples. Firstly, freeze-dried samples were mixed with KBr at a ratio of 1:100 and pressed into transparent thin pellets. The spectral acquisition range was 4000 to 400 cm^−1^, with a resolution of 8 cm^−1^ and 32 scans performed [21]. Background subtraction was conducted prior to sample measurement.

### 2.11. X-Ray Diffraction (XRD)

XRD was conducted according to the method of Ding et al. [22] with some modifications. Freeze-dried samples were placed on a glass slide and analyzed using an X-ray diffractometer (Rigaku, UItmia IV, Akishima, Japan). The measurement range was 5° to 80°, with a scanning speed of 4°/min and a step size of 0.2°. The light source was Cu-Kα radiation, operating at a tube voltage of 40 kV and a current of 40 mA.

### 2.12. X-Ray Photoelectron Spectroscopy (XPS)

High-resolution XPS (Nexsa, Thermo SCIENTIFIC, Waltham, MA, USA) scans of Zn2p in hydrogels were conducted to analyze the changes in the binding energies of these elements [23]. The specific procedure was as follows: an appropriate amount of hydrogel sample was adhered to the sample holder and placed in the sample chamber of the Thermo Scientific K-Alpha XPS instrument. The spot size was set to 400 μm, with a working voltage of 12 kV and a filament current of 6 mA. For full-spectrum scanning, the pass energy was 150 eV with a step size of 1 eV. For high-resolution (narrow-spectrum) scanning, the pass energy was 50 eV with a step size of 0.1 eV.

### 2.13. Statistical Analysis

SPSS 27.0 software (SPSS Inc., Chicago, IL, USA) was used to analyze results. Data was presented as means ± standard deviation (SD). Analysis of variance (ANOVA) was performed by the method of the least significant difference (LSD) at the significance level *p* < 0.05. Data was plotted using Origin Pro 2021 (Origin Lab Corporation, Northampton, MA, USA).

## 3. Results

### 3.1. Analysis of Zn^2+^ LC

**Figure 2:**
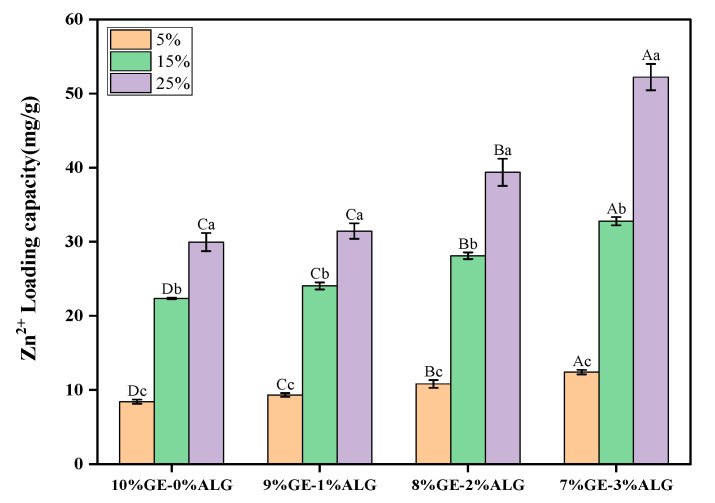
Zinc loading capacity of different hydrogels. 10%GE-0%ALG, 9%GE-1%ALG, 8%GE-2%ALG, 7%GE-3%ALG represent that the ratio of gelatin to sodium alginate in the hydrogel is 10:0, 9:1, 8:2, and 7:3, respectively; 5%, 15%, 25% represent the concentrations of zinc sulfate solution. Different uppercase letters indicate significant differences between groups, while different lowercase letters indicate significant differences within groups.

As shown in Figure 2, Zn^2+^ was successfully loaded into hydrogels by the soaking method. As the concentration of zinc sulfate solution increased, the LC of both pure gelatin hydrogel and xGE-yALG hydrogels increased significantly. Additionally, the Zn^2+^ LC of hydrogels with sodium alginate was significantly higher than that of pure gelatin hydrogels, especially when the concentration of zinc sulfate solution was above 5 wt%, indicating that the incorporation of sodium alginate further enhanced the Zn^2+^ LC. This enhancement is likely due to the crosslinking of sodium alginate with Zn^2+^ [24], which facilitated the incorporation of Zn^2+^. Specifically, the 7%GE-3%ALG hydrogel achieved the maximum LC of 52 mg/g after soaking in a 25 wt% zinc sulfate solution.

### 3.2. Analysis of Texture Profile

**Figure 3:**
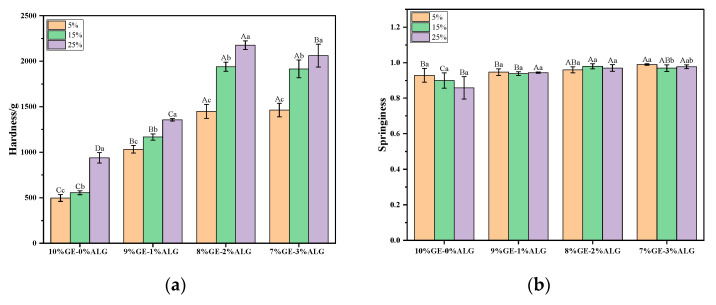
Texture profile of different zinc-loaded hydrogels. (**a**) Hardness and (**b**) springiness. 10%GE-0%ALG, 9%GE-1%ALG, 8%GE-2%ALG, and 7%GE-3%ALG represent that the ratio of gelatin to sodium alginate in the hydrogel is 10:0, 9:1, 8:2, and 7:3, respectively; 5%, 15%, 25% represent the concentration of zinc sulfate solution. Different uppercase letters indicate significant differences between groups, while different lowercase letters indicate significant differences within groups.

Hardness represents the force required for the hydrogel to deform to a certain extent, which could characterize the structure of a hydrogel. As shown in Figure 3a, increasing the concentration of zinc sulfate solution significantly increased the hardness of zinc-loaded hydrogels without or with different ratios of sodium alginate, indicating that the zinc sulfate solution soaking method strengthened the hydrogel structure. Cao et al. [25] also reported similar findings, observing that the gelatin hydrogel exhibited enhanced tensile and compressive properties after soaking in a zinc sulfate solution. They attributed this phenomenon to the Hofmeister effect, where SO_4_^2−^ enhances hydrogen bonding between polymer segments, resulting in a more compact internal network and increased crosslinking density. Simultaneously, introducing sodium alginate was also beneficial to strengthening the hydrogel structure. The hardness of zinc-loaded hydrogels containing sodium alginate was always higher than that of zinc-loaded hydrogels without sodium alginate, and when the ratio of gelatin to sodium alginate was 8:2, the zinc-loaded hydrogel exhibited the maximum hardness value. However, further increasing the ratio of sodium alginate was not conducive to the improvement of hardness, which may be due to the high concentration of sodium alginate affecting its compatibility with gelatin.

Figure 3b shows that the springiness of zinc-loaded hydrogels without sodium alginate gradually decreased upon increasing the zinc sulfate concentration, while the springiness of zinc-loaded hydrogels containing different proportions of sodium alginate remained consistently high, with values above 0.9, indicating that adding sodium alginate resulted in a tighter network structure and better springiness.

### 3.3. Analysis of Rheological Properties

In frequency sweep mode, continuously recording the storage modulus (G’) and loss modulus (G″) during testing allows for the analysis of the viscoelasticity and network structure of hydrogels [15].

**Figure 4:**
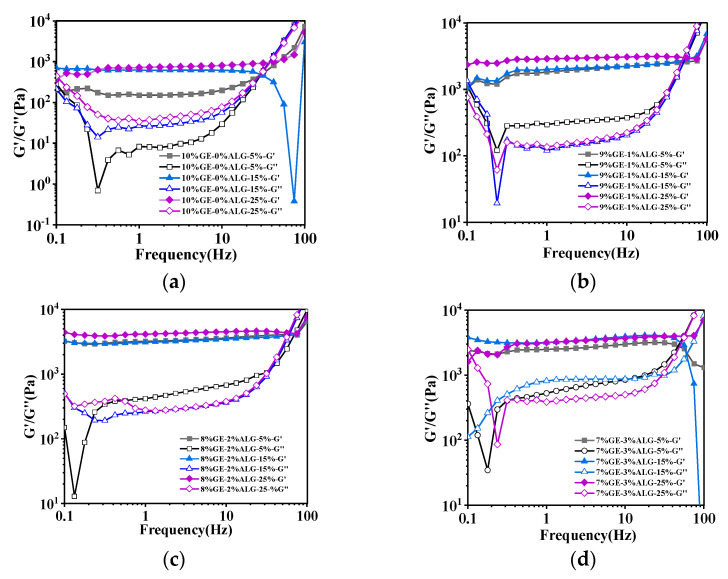
Rheological properties of different zinc-loaded hydrogels. (**a**) G′ and G″ of 10%GE-0%ALG hydrogels soaking in 5%, 15%, and 25% concentration of zinc sulfate solution; (**b**) G′ and G″ of 9%GE-1%ALG hydrogels soaking in 5%, 15%, and 25% concentration of zinc sulfate solution; (**c**) G′ and G″ of 8%GE-2%ALG hydrogels soaking in 5%, 15%, and 25% concentration of zinc sulfate solution; (**d**) G′ and G″ of 7%GE-3%ALG hydrogels soaking in 5%, 15%, and 25% concentration of zinc sulfate solution. 10%GE-0%ALG, 9%GE-1%ALG, 8%GE-2%ALG, 7%GE-3%ALG represent that the ratio of gelatin to sodium alginate in the hydrogel is 10:0, 9:1, 8:2, and 7:3, respectively.

Figure 4 shows that the value of G′ was consistently greater than that of G″ for all zinc-loaded hydrogels within the frequency range of 0.1 Hz to 100 Hz, demonstrating that these zinc-loaded hydrogels were mainly dominated by elastic properties [23]. Additionally, the G′ and G″ of gelatin hydrogel without sodium alginate both increased with the increase in zinc sulfate concentration, indicating that the hydrogel structure gradually strengthened with the increase in zinc sulfate concentration. This is mainly related to the additional crosslinking of gelatin molecular chains induced by SO_4_^2−^ [15]. After incorporating sodium alginate, the G′ and G″ of the zinc-loaded hydrogels further increased. This enhancement is likely due to the crosslinking between sodium alginate and Zn^2+^, as well as the interaction between sodium alginate and gelatin, resulting in a more compact and rigid network structure. Specifically, when the ratio of gelatin to sodium alginate was 8:2, the zinc-loaded hydrogel exhibited the maximum G′ value, suggesting the formation of stronger network structure in hydrogel compared to other zinc-loaded hydrogels [18]. Similarly, when the proportion of sodium alginate continued to increase, the G′ of the hydrogel slightly decreased as shown in Figure 4d, which aligns with the results for hardness.

Notably, as shown in Figure 4, when the frequency gradually increased, the G′ and G″ curves of the prepared zinc-loaded hydrogel intersected, which indicates that the structure of the hydrogel is destroyed at high frequencies. The crossing points of the G′ and G″ curves are summarized in Table 2. Obviously, the frequency corresponding to the intersection of G′ and G″ curves of the zinc-loaded hydrogels containing sodium alginate was much higher than that of the zinc-loaded hydrogels without sodium alginate. Moreover, when the ratio of gelatin to sodium alginate was 8:2, the frequency at the intersection of the G′ and G″ curves of the zinc-loaded hydrogel was generally higher, once again indicating the relatively strong structure of these hydrogels.

### 3.4. Analysis of Melting Temperature and Gelling Temperature

**Figure 5:**
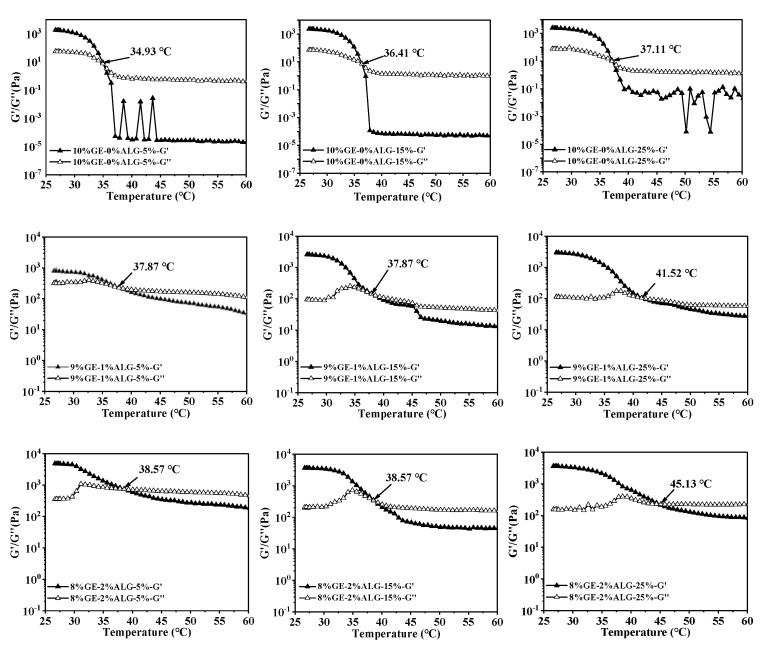

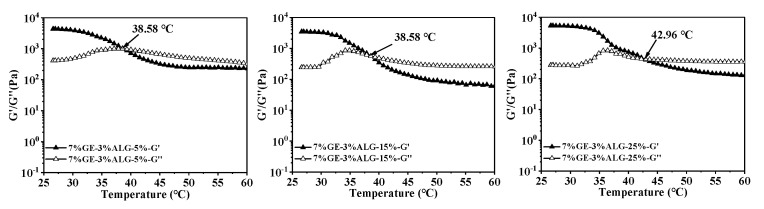
Melting temperature of different hydrogels. 10%GE-0%ALG, 9%GE-1%ALG, 8%GE-2%ALG, 7%GE-3%ALG represent that the ratio of gelatin to sodium alginate in the hydrogel is 10:0, 9:1, 8:2, and 7:3, respectively; 5%, 15%, 25% represent the concentration of zinc sulfate solution.

In thermo-reversible hydrogels, the structure weakens with temperature increases, leading to a decrease in both G′ and G″. The intersection of the G′ and G″ curves is defined as the melting temperature of the hydrogel, at which the hydrogel changes from a solid-like gel state to a liquid state [26]. Figure 5 shows that as temperature increased, the G′ and G″ of zinc-loaded hydrogels without sodium alginate decreased rapidly, indicating an unstable gel network prone to weakening. As the concentration of zinc sulfate solution increased from 5 wt% to 25 wt%, the melting temperature of zinc-loaded hydrogel without sodium alginate increased from 34.93 °C to 37.11 °C (shown in Table 2). A similar trend was observed in the zinc-loaded hydrogels containing sodium alginate, where the melting temperature rose with the increase in zinc sulfate concentration. This suggested that a high concentration of zinc sulfate solution is conducive to improving the thermal stability of hydrogels. Qiao, Wang, Zhang, & Yao [27] observed that the melting temperature of gelatin significantly increased after immersion in a potassium sulfate solution, which was attributed to the promotion of triple helix formation by SO_4_^2−^, leading to an enhanced number of triple helices and tighter aggregation within the gelatin structure. Furthermore, adding sodium alginate slowed down the decline rate of G′ and G″ of the hydrogels, causing the hydrogels to melt at a higher temperature, representing that sodium alginate strengthened hydrogel structure [28]. This might be related to the electrostatic interactions between sodium alginate and gelatin, as well as between sodium alginate and Zn^2+^.

**Figure 6:**
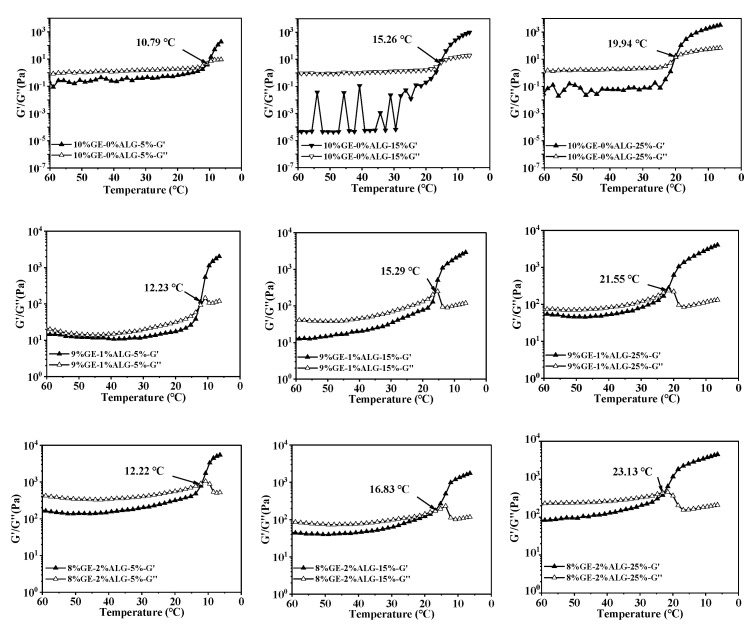

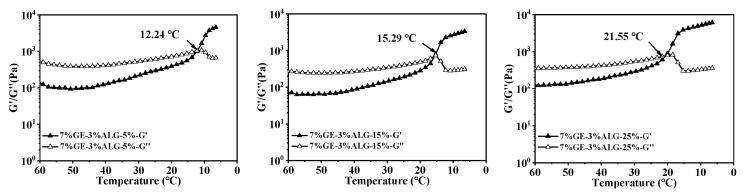
Gelling temperature of different hydrogels. 10%GE-0%ALG, 9%GE-1%ALG, 8%GE-2%ALG, 7%GE-3%ALG represent that the ratio of gelatin to sodium alginate in the hydrogel is 10:0, 9:1, 8:2, and 7:3, respectively; 5%, 15%, 25% represent the concentration of zinc sulfate solution.

When the temperature drops, the increased G′ and G″ will come to an intersection, and the intersection point is defined as the gelling temperature of hydrogels, at which the hydrogel changes from a liquid state to a solid-like gel state. Figure 6 shows that as the concentration of zinc sulfate increased, the gelling temperature of all hydrogels gradually increased, suggesting that the presence of zinc sulfate enables gelatin solution and gelatin-sodium alginate solution to form a hydrogel at higher temperatures. The specific values of the gelling temperature are shown in Table 2. According to Qiao et al. [27], the gelling temperature of hydrogels increases linearly with the hydration entropy of anions in the aqueous solution. Strong hydration of SO_4_^2−^ polarizes water molecules in the first hydration layer of gelatin, resulting in the dehydration of hydrophilic biopolymers, causing the binding of individual polymer chains and promoting the gelation of gelatin solution. Additionally, the gelling temperature also increased after introducing sodium alginate, with the highest gelling temperature of 23.13 °C observed for the 8%GE-2%ALG-25% hydrogel. This also indicated that when gelatin and sodium alginate are in an appropriate proportion, the interaction between gelatin and sodium alginate, as well as the presence of zinc sulfate are conducive to the formation of the hydrogel structure.

### 3.5. Analysis of pH Sensitivity

**Figure 7:**
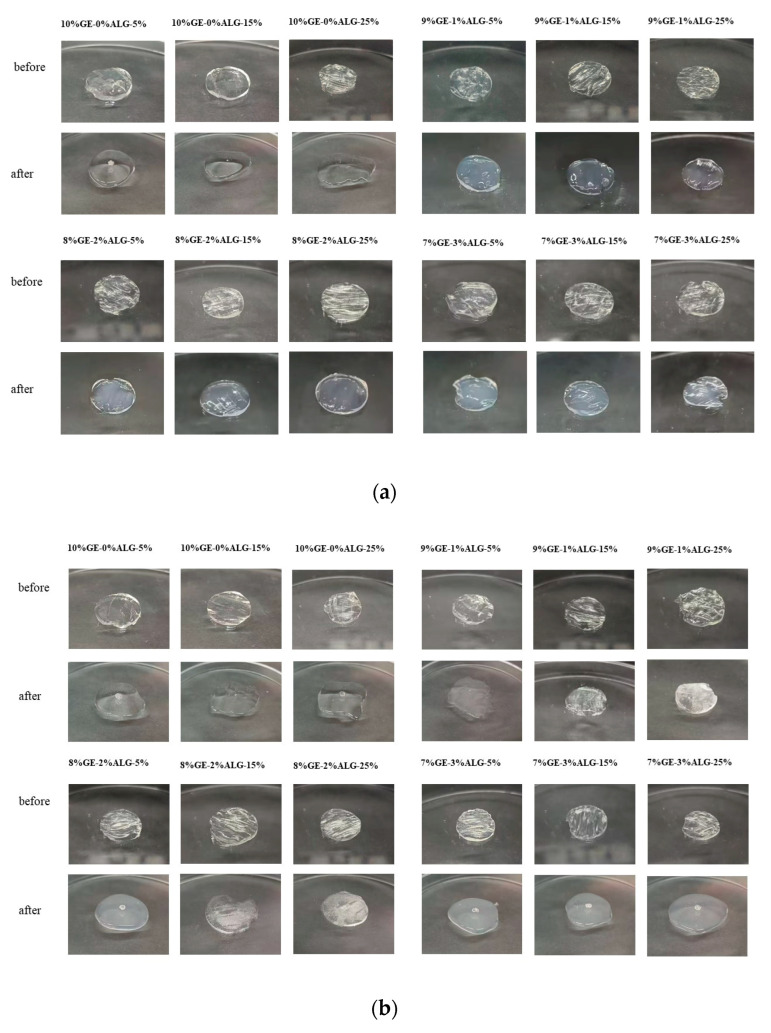
pH sensitivity of different hydrogels. Morphology images of hydrogels before and after 12 h swelling in PBS solutions at (**a**) pH 2 and (**b**) pH 7. 10%GE-0%ALG, 9%GE-1%ALG, 8%GE-2%ALG, 7%GE-3%ALG represent that the ratio of gelatin to sodium alginate in the hydrogel is 10:0, 9:1, 8:2, and 7:3, respectively; 5%, 15%, 25% represent the concentration of zinc sulfate solution.

The pH sensitivity of hydrogels significantly influences the controlled release behavior of nutrients encapsulated within [23]. As shown in Figure 7, the zinc-loaded hydrogels presented different morphologies after swelling in PBS solution with pH of 2 and 7.

At pH 2, the hydrogel without sodium alginate completely transformed from a gel state to a liquid state, while hydrogels containing sodium alginate basically maintained a complete gel state, which indicates the stable structure of hydrogels containing sodium alginate under acidic conditions. This phenomenon can be attributed to the reduced ionization of carboxyl groups in sodium alginate under acidic conditions, which lowers the swelling of the hydrogel [29]. At pH 7, hydrogels without sodium alginate also completely transform from a gel state to a liquid state, while hydrogels containing sodium alginate undergo varying degrees of morphological transformation from a gel state to a liquid state. Notably, when the ratio of gelatin to sodium alginate was 7:3, the 7%GE-3%ALG-5% hydrogel, 7%GE-3%ALG-15% hydrogel, and 7%GE-3%ALG-25% hydrogel all completely transformed from a gel state to a liquid state in the PBS solution at pH 7, indicating that increasing the sodium alginate proportion reduced the structural stability of zinc-loaded hydrogels under neutral conditions. This is likely due to the enhanced ionization of carboxyl groups under neutral conditions, increasing the hydrophilicity of the hydrogel and promoting water absorption and subsequent morphological transformation [30]. These findings indicate that hydrogels with a ratio of gelatin to sodium alginate of 7:3 exhibited a certain pH response behavior, characterized by less swelling in acidic environments, maintaining a complete gel state to protect the encapsulated Zn^2+^, while fully swelling and transforming into a liquid state under neutral or alkaline conditions to promote the release of Zn^2+^ [31].

### 3.6. Analysis of Microscopic Morphology

**Figure 8:**
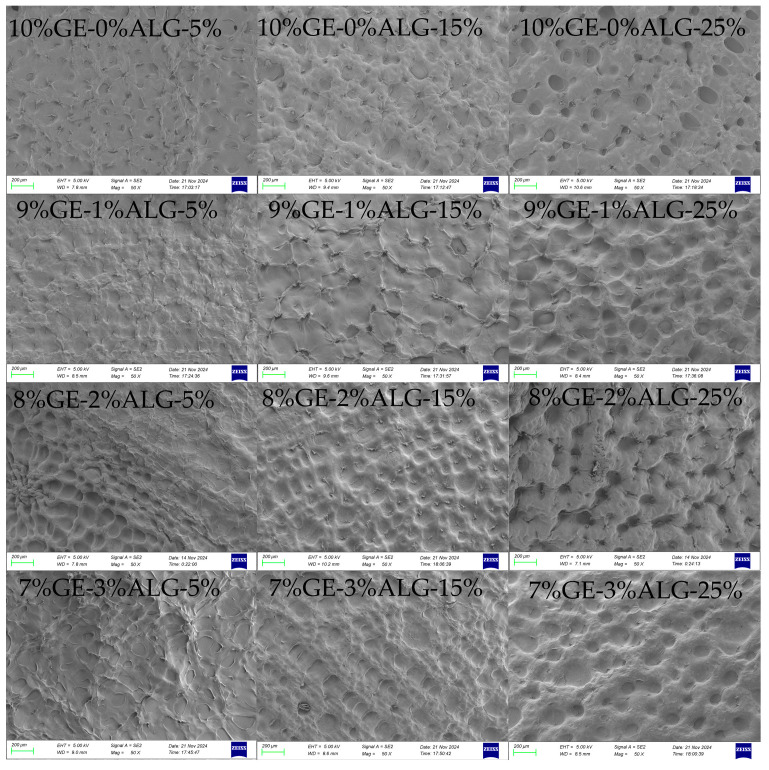
SEM images of the cross-sectional morphology of different hydrogels. 10%GE-0%ALG, 9%GE-1%ALG, 8%GE-2%ALG, 7%GE-3%ALG represent that the ratio of gelatin to sodium alginate in the hydrogel is 10:0, 9:1, 8:2, and 7:3, respectively; 5%, 15%, 25% represent the concentration of zinc sulfate solution.

The microstructure of hydrogels is closely related to their macroscopic physical properties [19]. As shown in Figure 8, all zinc-loaded hydrogels exhibited a dense porous crosslinked structure. When the concentration of zinc sulfate solution increased, the porous structure of all hydrogels became more obvious and the pores deepened. This might be because the high concentration of zinc sulfate solution induced the formation of denser crosslinking in the hydrogel [15]. After introducing sodium alginate, no obvious change was observed in the microscopic morphology of hydrogels except for in the 8%GE-2%ALG-25% hydrogel. The morphology of the 8%GE-2%ALG-25% hydrogel displayed larger and deeper honeycomb-like pore structures compared with other hydrogels, which might be the reason for the high hardness and G′ of the 8%GE-2%ALG-25% hydrogel.

### 3.7. Analysis of FTIR Spectrum

**Figure 9:**
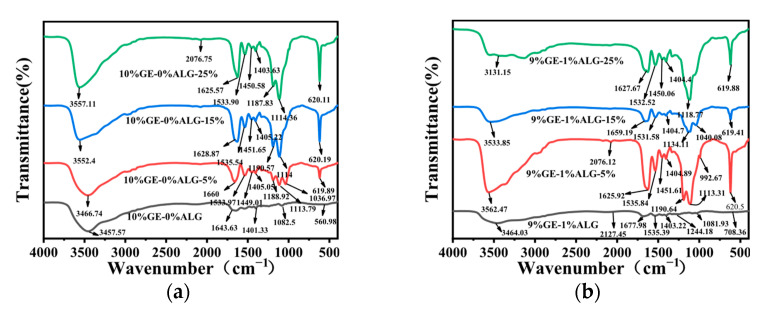

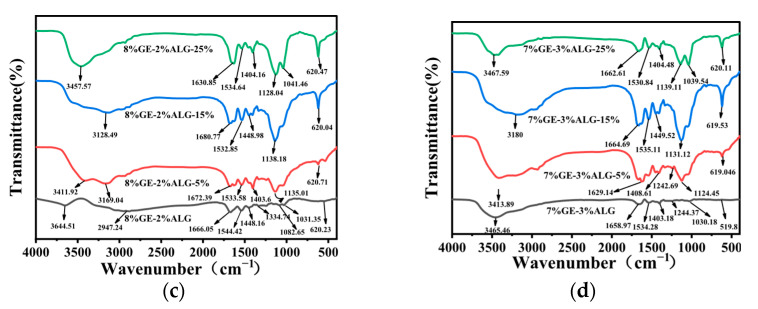
FTIR spectra of different hydrogels. (**a**) 10%GE-0%ALG hydrogels soaking in 5%, 15%, and 25% concentration of zinc sulfate solution; (**b**) 9%GE-1%ALG hydrogels soaking in 5%, 15%, and 25% concentration of zinc sulfate solution; (**c**) 8%GE-2%ALG hydrogels soaking in 5%, 15%, and 25% concentration of zinc sulfate solution; (**d**) 7%GE-3%ALG hydrogels soaking in 5%, 15%, and 25% concentration of zinc sulfate solution. 10%GE-0%ALG, 9%GE-1%ALG, 8%GE-2%ALG, 7%GE-3%ALG represent that the ratio of gelatin to sodium alginate in the hydrogel is 10:0, 9:1, 8:2, and 7:3, respectively.

To explore the interactions among gelatin, sodium alginate, and Zn^2+^, FTIR spectroscopy was conducted. As shown in Figure 9a, 10%GE-0%ALG hydrogel exhibited a characteristic absorption peak at 3457.57 cm^−1^, attributed to the stretching vibrations of O-H and N-H groups associated with intramolecular hydrogen bonding [15]. The peaks at 1643.63 cm^−1^ and 1401.33 cm^−1^ correspond to the stretching vibration of the amide I band (C=O) and the bending vibration of C-H, respectively [32]. Figure S1 showed that the characteristic peaks of sodium alginate include a broad peak at 3431.74 cm^−1^ attributed to the O-H stretching vibration [33], an asymmetric stretching vibration of the carbonyl group in COO- at 1603.91 cm^−1^, and a symmetric stretching vibration of the carbonyl group in COO- at 1417.02 cm^−1^ [34]. The characteristic peaks of heptahydrate zinc sulfate include a Zn-O stretching vibration band at 1123.04 cm^−1^, a Zn-O bending vibration band associated with water molecule absorption at 616.42 cm^−1^, and a Zn-O-Zn bending vibration band at 527.27 cm^−1^ [35].

As shown in Figure 9b–d, significant changes were observed in the xGE-yALG hydrogels. Specifically, the peak at 3457.57 cm^−1^ shifted to 3464.03 cm^−1^, 3644.51 cm^−1^, and 3465.46 cm^−1^ in the 9%GE-1%ALG hydrogel, 8%GE-2%ALG hydrogel, and 7%GE-3%ALG hydrogel, suggesting the formation of hydrogen bonds between the hydroxyl groups of sodium alginate and the amino groups of gelatin [36]. Additionally, the amide I band peak at 1643.63 cm^−1^ shifted to 1677.98 cm^−1^, 1666.05 cm^−1^ and 1658.97 cm^−1^, and a new peak appeared at 1535.39 cm^−1^, 1544.42 cm^−1^ and 1534.28 cm^−1^ in the 9%GE-1%ALG hydrogel, 8%GE-2%ALG hydrogel, and 7%GE-3%ALG hydrogel, corresponding to the amide II band. These shifts indicated potential electrostatic interactions between the positively charged amino acid residues in gelatin and the negatively charged carboxyl groups in sodium alginate [37]. 

After loading Zn^2+^, the absorption peak of the zinc-loaded hydrogel without sodium alginate at 3457.57 cm^−1^ shifted to 3466.74 cm^−1^, 3552.4 cm^−1^, and 3557.11 cm^−1^, as shown in Figure 9a, with increased peak intensity as the concentration of zinc sulfate increased. This suggests that the electron cloud density of N-H has been enhanced due to the formation of N-Zn coordination bonds [35]. For the zinc-loaded hydrogels containing sodium alginate shown in Figure 9b–d, the absorption peaks of the amide A and I bands also shifted with increasing zinc sulfate concentration. Notably, the COO− absorption band of sodium alginate in the zinc-loaded hydrogels shifts to a higher wavenumber, likely due to the formation of COO-Zn complexes [24]. This shift indicates chelation between the carboxyl groups of sodium alginate and Zn^2+^. Furthermore, the Zn-O-Zn bending vibration band of heptahydrate zinc sulfate at 616.42 cm^−1^ also exhibits a shift to a higher wavenumber. These observations collectively suggest that Zn^2+^ has crosslinked with the hydrogel [35].

### 3.8. Analysis of XRD Spectroscopy

**Figure 10:**
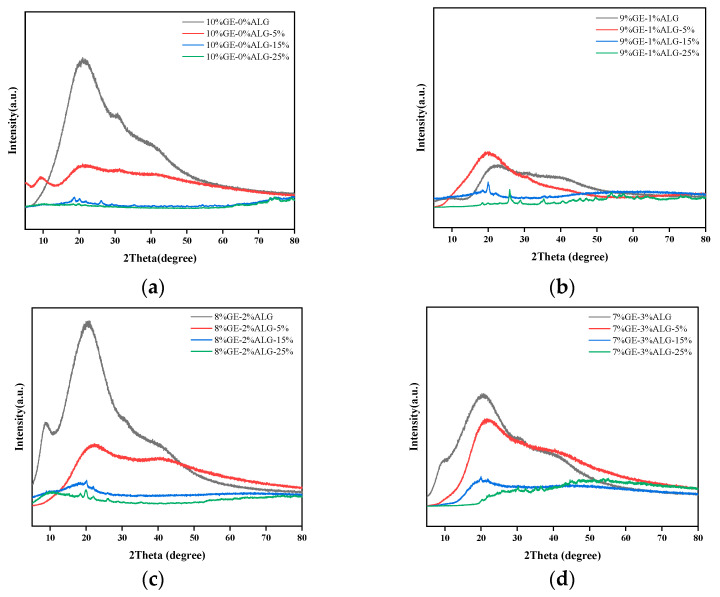
XRD patterns of different hydrogels. (**a**) 10%GE-0%ALG hydrogels soaking in 5%, 15%, and 25% concentration of zinc sulfate solution; (**b**) 9%GE-1%ALG hydrogels soaking in 5%, 15%, and 25% concentration of zinc sulfate solution; (**c**) 8%GE-2%ALG hydrogels soaking in 5%, 15%, and 25% concentration of zinc sulfate solution; (**d**) 7%GE-3%ALG hydrogels soaking in 5%, 15%, and 25% concentration of zinc sulfate solution. 10%GE-0%ALG, 9%GE-1%ALG, 8%GE-2%ALG, 7%GE-3%ALG represent that the ratio of gelatin to sodium alginate in the hydrogel is 10:0, 9:1, 8:2, and 7:3, respectively.

The crystal structure of zinc-loaded hydrogels was analyzed using X-ray diffraction (XRD). As shown in Figure 10a, a broad diffraction peak was observed at approximately 2θ = 20° for the 10%GE-0%ALG hydrogel, indicating its amorphous structure [38]. When the hydrogel without sodium alginate was immersed in zinc sulfate solution, the intensity of the characteristic peak around 20° dropped sharply, which represents a reduction in the amorphous structure of the hydrogel. According to the literature, highly hydrophilic SO_4_^2−^ will competitively bind water molecules with gelatin, enhancing the hydrophobic interaction between gelatin chains, making the gelatin chains tend to approach each other, and strengthening the chain bundles between the molecular chains [25,32]. This might be the reason why the amorphous regions in the zinc-loaded hydrogel decrease after soaking in zinc sulfate solution. In addition, after loading Zn^2+^, the combination of Zn^2+^ with functional groups such as hydroxyl, carboxyl, and amino groups in gelatin molecules may also lead to changes in the amorphous structure of the hydrogel. The specific binding mode of Zn^2+^ needs to be further explored through subsequent XPS experiments.

After incorporating sodium alginate, the intensity of the characteristic peak of xGE-yALG hydrogels decreased slightly, as shown in Figure 10b–d. This reduction is likely due to electrostatic interactions between the carboxyl groups of sodium alginate and the amino groups of gelatin, which interfere with the amorphous structure to a certain extent [37]. Notably, after adding sodium alginate, a new diffraction peak appeared in the xGE-yALG hydrogels near 2θ = 9°, indicating that a regular crystal structure appeared in the xGE-yALG hydrogels [11], which may be the reason why the hardness and the melting temperature of hydrogels containing sodium alginate are higher than that of hydrogels without sodium alginate. After loading Zn^2+^ through the zinc sulfate solution soaking method, the intensity of the diffraction peak at 20° also decreased sharply in xGE-yALG hydrogels, especially when the concentration of zinc sulfate solution was 15 wt% and 25 wt%, which may be related the SO_4_^2−^-enhanced hydrophobic interaction and the binding of Zn^2+^ to the hydrogel matrix. In addition, the diffraction peak of the 8%GE-2%ALG-25% hydrogel completely disappeared at 2θ = 20°, while an obvious diffraction peak appeared at 2θ = 9°, which corresponds to the crystal structure of the 8%GE-2%ALG-25% hydrogel, and also exactly corresponds to the relatively stable gel structure of the 8%GE-2%ALG-25% hydrogel.

### 3.9. Analysis of XPS

XPS can provide the chemical composition of zinc-loaded hydrogels and determine the bonding type of Zn^2+^ in zinc-loaded hydrogels [39].

**Figure 11:**
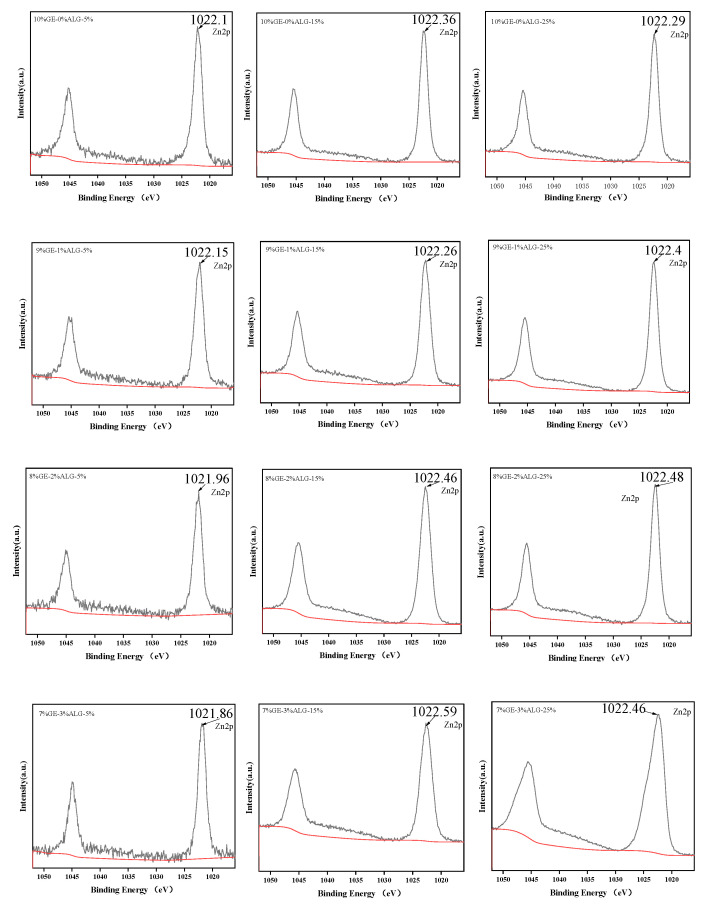
High-resolution XPS spectra for Zn2p of different hydrogels. 10%GE-0%ALG, 9%GE-1%ALG, 8%GE-2%ALG, 7%GE-3%ALG represent that the ratio of gelatin to sodium alginate in the hydrogel is 10:0, 9:1, 8:2, and 7:3, respectively; 5%, 15%, 25% represent the concentration of zinc sulfate solution.

As shown in Figure 11, the binding energy of zinc in all zinc-loaded hydrogels was around 1022 eV, which corresponds to the Zn-O peak, indicating the existence of Zn-O bonds in zinc-loaded hydrogels [40]. The results of the XPS suggest that Zn^2+^ formed coordination bonds with oxygen atoms.

## 4. Conclusions

In summary, gelatin-based zinc-loaded hydrogels were successfully constructed with the assistance of sodium alginate and a zinc sulfate solution soaking method. The 7%GE-3%ALG hydrogel achieved the maximum zinc loading capacity of 52 mg/g after soaking in a 25 wt% zinc sulfate solution. Adding sodium alginate not only strengthened the hydrogel structure but also endowed the hydrogel with a certain pH sensitivity. When the ratio of gelatin to sodium alginate was 7:3, the hydrogel maintained its structural integrity in a pH 2 environment, but changed from a gel state to a liquid state in a pH 7 environment. Subsequent experiments will explore the simulated gastrointestinal digestion and the kinetics of Zn^2+^ release.

## Figures and Tables

**Table 1:** The formula of different hydrogels.

Hydrogel Sample	GE (wt%)	ALG (wt%)	Zinc Sulfate Solutions (wt%)
10%GE-0%ALG-5%	10	0	5
10%GE-0%ALG-15%	10	0	15
10%GE-0%ALG-25%	10	0	25
9%GE-1%ALG-5%	9	1	5
9%GE-1%ALG-15%	9	1	15
9%GE-1%ALG-25%	9	1	25
8%GE-2%ALG-5%	8	2	5
8%GE-2%ALG-15%	8	2	15
8%GE-2%ALG-25%	8	2	25
7%GE-3%ALG-5%	7	3	5
7%GE-3%ALG-15%	7	3	15
7%GE-3%ALG-25%	7	3	25

Note: GE and ALG represent gelatin and sodium alginate, respectively.

**Table 2:** The crossing points of G′ and G″ curves under frequency scanning and temperature scanning.

Hydrogel Sample	Frequency (Hz)	Heating (°C)	Cooling (°C)
10%GE-0%ALG-5%	28	34.93	10.79
10%GE-0%ALG-15%	29	36.41	15.26
10%GE-0%ALG-25%	37.5	37.11	19.94
9%GE-1%ALG-5%	51	37.87	12.23
9%GE-1%ALG-15%	51.5	37.87	15.29
9%GE-1%ALG-25%	50.5	41.52	21.55
8%GE-2%ALG-5%	69.5	38.57	12.22
8%GE-2%ALG-15%	59	38.57	16.83
8%GE-2%ALG-25%	58.5	45.13	23.13
7%GE-3%ALG-5%	47	38.58	12.24
7%GE-3%ALG-15%	62	38.58	15.29
7%GE-3%ALG-25%	57.5	42.96	21.55

## Data Availability

The authors declare that the data supporting the findings of this study are available within the paper. Should any raw data files be needed in another format they are available from the corresponding author upon reasonable request.

## Supplementary Materials

The following supporting information can be downloaded at: https://www.mdpi.com/article/10.3390/foods14213642/s1, Figure S1: FTIR spectra of zinc sulfate heptahydrate and sodium alginate.

## References

[1] Xie N., Huang J., Li B., Cheng J., Wang Z., Yin J., Yan X. (2015). Affinity purification and characterisation of zinc chelating peptides from rapeseed protein hydrolysates: Possible contribution of characteristic amino acid residues. Food Chem..

[2] Duan M., Li T., Liu B., Yin S., Zang J., Lv C., Zhao G., Zhang T. (2021). Zinc nutrition and dietary zinc supplements. Crit. Rev. Food Sci. Nutr..

[3] Wang M., Phadke M., Packard D., Yadav D., Gorelick F. (2020). Zinc: Roles in pancreatic physiology and disease. Pancreatology.

[4] Qiao F., Yu X., Tie S., Chen Y., Hou S., Tan M. (2021). Zinc delivery system constructed from food-borne nanoparticles derived from *Undaria pinnatifida*. Food Funct..

[5] Udechukwu M.C., Collins S.A., Udenigwe C.C. (2016). Prospects of enhancing dietary zinc bioavailability with food-derived zinc-chelating peptides. Food Funct..

[6] Jiang W., Zhai S., Zhu L., Bai Y., Li J., Li J. (2024). Protein/polysaccharide based oral delivery system for precise targeting of polyphenols and carotenoids. Food Biosci..

[7] Irfan J., Hussain M.A., Haseeb M.T., Ali A., Farid-ul-Haq M., Tabassum T., Hussain S.Z., Hussain I., Naeem-ul-Hassan M. (2021). A pH-sensitive, stimuli-responsive, superabsorbent, smart hydrogel from psyllium (*Plantago ovata*) for intelligent drug delivery. RSC Adv..

[8] Singh A., Kar A.K., Singh D., Verma R., Shraogi N., Zehra A., Gautam K., Anbumani S., Ghosh D., Patnaik S. (2022). pH-responsive eco-friendly chitosan modified cenosphere/alginate composite hydrogel beads as carrier for controlled release of imidacloprid towards sustainable pest control. Chem. Eng. J..

[9] Gang F., Li R., Dang Z., Meng X., Zhang J., Sun X. (2025). High-stretchable, transparent, degradable and anti-drying gelatin hydrogel for mango preservation. LWT-Food Sci. Technol..

[10] Yang X., Tan Z., Zhao W., Zheng Y., Ling S., Guo X., Dong X. (2025). Molecular interactions and gel network modulation in ionic polysaccharide-gelatin hydrogels for improved texture of skipjack tuna products. Food Chem..

[11] Chen H., Lan X., Guan X., Luo R., Zhang Q., Ren H., Xu Z., Tang J. (2024). Comparative study on the effects of chitosan, carrageenan, and sodium alginate on the film-forming properties of fish skin gelatin. LWT Food Sci. Technol..

[12] Hao Y., Zhou M., Chen R., Mao X., Huang W. (2023). A bioinspired hydrogel carrier with pH/redox dual responsiveness for effective protection and intestinal targeted delivery of probiotics. J. Food Eng..

[13] Qiao S., Chen W., Zheng X., Ma L. (2024). Preparation of pH-sensitive alginate-based hydrogel by microfluidic technology for intestinal targeting drug delivery. Int. J. Biol. Macromol..

[14] Pereira R.N., Rodrigues R.M., Altinok E., Ramos Ó.L., Malcata F.X., Maresca P., Ferrari G., Teixeira J.A., Vicente A.A. (2017). Development of iron-rich whey protein hydrogels following application of ohmic heating-effects of moderate electric fields. Food Res. Int..

[15] Chen H., Wu D., Ma W., Wu C., Tian Y., Wang S., Du M. (2021). Strong fish gelatin hydrogels enhanced by carrageenan and potassium sulfate. Food Hydrocoll..

[16] Yaman M., Kaya G. (2005). Speciation of iron (II) and (III) by using solvent extraction and flame atomic absorption spectrometry. Anal. Chim. Acta.

[17] Xie Y., Zhao K., Peng J., Jiang L., Shu W., Huang Y., Liu Q., Luo W., Yuan Y. (2025). Effect of β-glucan on the gelling properties of unwashed silver carp surimi gel: Insights into molecular interactions between different sources of β-glucan and myofibrillar protein. Food Res. Int..

[18] Chen H., Wu D., Ma W., Wu C., Liu J., Du M. (2022). Strong fish gelatin hydrogels double crosslinked by transglutaminase and carrageenan. Food Chem..

[19] Wang L., Dong Y., Jiang L., Zhang Y., Sui X. (2025). The Hofmeister series: Anion effect on microbial transglutaminase cross-linked soybean protein isolate hydrogels. Food Chem..

[20] Yan W., Jia X., Zhang Q., Chen H., Zhu Q., Yin L. (2021). Interpenetrating polymer network hydrogels of soy protein isolate and sugar beet pectin as a potential carrier for probiotics. Food Hydrocoll..

[21] Xu J., Yan S., Qi B., Jiang L. (2025). New insights into the cross-linking mechanism of soybean protein-based double dynamic cross-linking hydrogels for the controlled delivery of curcumin. Food Res. Int..

[22] Ding X., Xu Y., Wang Y., Xie L., Liang S., Li D., Wang Y., Wang J., Zhan X. (2022). Carboxymethyl konjac glucomannan-chitosan complex nanogels stabilized double emulsions incorporated into alginate hydrogel beads for the encapsulation, protection and delivery of probiotics. Carbohydr. Polym..

[23] Hu X., Wang Y., Zhang L., Xu M. (2020). Formation of self-assembled polyelectrolyte complex hydrogel derived from salecan and chitosan for sustained release of Vitamin C. Carbohydr. Polym..

[24] Zhang X., Miao F., Niu L., Wei Y., Hu Y., Lian X., Zhao L., Chen W., Huang D. (2021). Berberine carried gelatin/sodium alginate hydrogels with antibacterial and EDTA-induced detachment performances. Int. J. Biol. Macromol..

[25] Cao G., Zhao L., Ji X., Peng Y., Yu M., Wang X., Li X., Ran F. (2023). “Salting out” in Hofmeister effect enhancing mechanical and electrochemical performance of Amide-based hydrogel electrolytes for flexible zinc-ion battery. Small.

[26] Torres M., Chenlo F., Moreira R. (2017). Thermal reversibility of kappa/iota-hybrid carrageenan gels extracted from *Mastocarpus stellatus* at different ionic strengths. J. Taiwan Inst. Chem. E.

[27] Qiao C., Wang X., Zhang J., Yao J. (2021). Influence of salts in the Hofmeister series on the physical gelation behavior of gelatin in aqueous solutions. Food Hydrocoll..

[28] Haug I.J., Draget K.I., Smidsrød O. (2004). Physical behaviour of fish gelatin-κ-carrageenan mixtures. Carbohyd. Polym..

[29] Tsai F.H., Kitamura Y., Kokawa M. (2017). Effect of gum arabic-modified alginate on physicochemical properties, release kinetics, and storage stability of liquid-core hydrogel beads. Carbohyd. Polym..

[30] Premjit Y., Pandey S., Mitra J. (2024). Encapsulation of probiotics in freeze-dried calcium alginate and κ-carrageenan beads using definitive screening design: A comprehensive characterisation and in vitro digestion study. Int. J. Biol. Macromol..

[31] Aycan D. (2024). Alginate/hyaluronic acid/gelatin ternary blended films as pH-sensitive drug carriers: In vitro ampicillin release and kinetic studies. Int. J. Biol. Macromol..

[32] He Q., Huang Y., Wang S. (2018). Hofmeister effect-assisted one step fabrication of ductile and strong gelatin hydrogels. Adv. Funct. Mater..

[33] Lopes S., Bueno L., De Aguiar F., Finkler C. (2017). Preparation and characterization of alginate and gelatin microcapsules containing *Lactobacillus rhamnosus*. An. Acad. Bras. Ciênc..

[34] Wang M., Zang Y., Hong K., Zhao X., Yu C., Liu D., An Z., Wang L., Yue W., Nie G. (2021). Preparation of pH-sensitive carboxymethyl cellulose/chitosan/alginate hydrogel beads with reticulated shell structure to deliver Bacillus subtilis natto. Int. J. Biol. Macromol..

[35] Sun R., Liu X., Yu Y., Miao J., Leng K., Gao H. (2021). Preparation process optimization, structural characterization and in vitro digestion stability analysis of Antarctic krill (*Euphausia superba*) peptides-zinc chelate. Food Chem..

[36] Zhang X., Liu K., Qin M., Lan W., Wang L., Liang Z., Li X., Wei Y., Hu Y., Zhao L. (2023). Abundant tannic acid modified gelatin/sodium alginate biocomposite hydrogels with high toughness, antifreezing, antioxidant and antibacterial properties. Carbohyd. Polym..

[37] Ni F., Luo X., Zhao Z., Yuan J., Song Y., Liu C., Huang M., Dong L., Xie H., Ren G. (2023). Enhancing viability of *Lactobacillus plantarum* encapsulated by alginate-gelatin hydrogel beads during gastrointestinal digestion, storage and in the mimic beverage systems. Int. J. Biol. Macromol..

[38] Liu J., Yong H., Liu Y., Qin Y., Kan J., Liu J. (2019). Preparation and characterization of active and intelligent films based on fish gelatin and haskap berries (*Lonicera caerulea* L.) extract. Food Packag. Shelf Life.

[39] Cieślik A., Shymborska Y., Tymetska S., Stetsyshyn Y., Bernasik A., Brzychczy-Włoch M., Drożdż K., Szajna K., Krok F., Budkowski A. (2025). Cell sheet engineering platforms integrating antibacterial and thermo-responsive functionalities: Cu-nanoparticle-loaded P4VP brushes for retinal cell sheet harvesting. Chem. Eng. J..

[40] Zheng S., Lin S., Xu Y., Cai X., Wang S. (2024). Antibacterial activity and virulence attenuation of peptides-zinc nanocomposite against *Vibrio alginolyticus*. Food Biosci..

