# Supplementary figures and images for: Gelatin-Based Zinc-Loaded Hydrogels Constructed with the Assistance of Sodium Alginate and Zinc Sulfate Solution Soaking Method
Source: Foods. 2025 Oct 24;14(21):3642. doi: 10.3390/foods14213642 (PMC12609939; doi:10.3390/foods14213642)

**Figure S1.** FTIR spectra of zinc sulfate heptahydrate and sodium alginate.

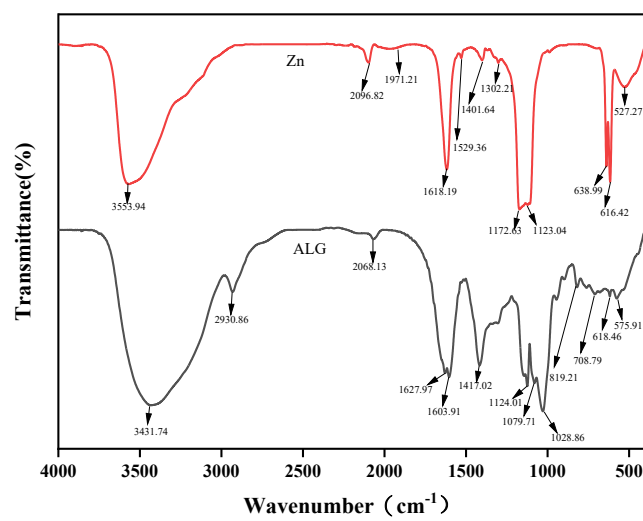

Supplement: Supplementary file 1 [file foods-14-03642-s001.zip › foods-3900228-supplementary.pdf]
